# Ex-vivo expanded human NK cells mediate cytotoxicity and cytokine release against allogeneic cancer cell line by direct recognition and antibody directed cellular cytotoxicity: therapeutic potential use of NK cells for blood and solid tumors

**DOI:** 10.1186/2051-1426-2-S1-P1

**Published:** 2014-02-24

**Authors:** Abdelhamid Liacini, Noureddine Berka, Faisal Khan

**Affiliations:** 1 Pathology & Laboratory Medicine, Histocompatibility & Immunogenetics Laboratory, King Fahad Specialist Hospital, Dammam, Saudi Arabia; 2 Pathology & Laboratory Medicine, Calgary Laboratory Services, Calgary, Alberta, Canada

## 

Implementation of NK cell therapy is faced by several obstacles: the small number of NK cells in peripheral blood and the difficulties regarding production of effective cytolytic NK cells. We carried out this pilot study to determine if NK cells could be expanded and their degranulation, cytokines release abilities in response to allogeneic tumor target by direct cytotoxitiy and by antibody-mediated cellular cytotoxicity can be achieved.

## Methods

MNCs cells from healthy donors were stimulated with K562. U293T cells were co-transduced with constructs encoding the membrane-bound form of IL-15 and human 4-1BBL. A 5-colour flow cytometry based estimation of cytotoxicity (expression of CD107a, a surrogate marker for degranulation) and cytokine (IFN-γ) production was performed for both CD56brightCD16neg regulatory and CD56dimCD16pos cytolytic NK cell subsets.

## Results

Cell expansion of NK cell is feasible and both degranulation and IFN-γ release were specifically triggered by cytolytic and regulatory NK cells against allogeneic tumor cell line K562. Importantly, expanded NK cells also mediated ADCC when cultured with PBMNCs in the presence of serum bearing HLA-Class I and II antibodies

## Conclusion

Large number of cytolytic and regulatory NK cells can be generated from MNCs in vitro suggesting the potential use of NK cell-based immunotherapy in tumors.

